# An SNP-based diagnostic method for *Brucella* S2 vaccine strain infections

**DOI:** 10.3389/fvets.2025.1570220

**Published:** 2025-06-19

**Authors:** Xingya Wang, Xiaowei Tian, Wanyang Li, Yuanchao Yang, Shuai Zhang, Hui Wang, Wanru Geng, Jingbo Zhai

**Affiliations:** ^1^School of Basic Medical Sciences, Inner Mongolia Minzu University, Tongliao, China; ^2^Department of Laboratory, Hulunbuir Second People’s Hospital, Hulunbuir, China; ^3^Department of Intensive Medicine, Affiliated Hospital of Inner Mongolia Minzu University, Tongliao, China; ^4^College of Clinical Medicine, Inner Mongolia Minzu University, Tongliao, China; ^5^Key Laboratory of Zoonose Prevention and Control at Universities of Inner Mongolia Autonomous Region, Tongliao, China; ^6^Brucellosis Prevention and Treatment Engineering Research Center of Inner Mongolia Autonomous Region, Tongliao, China

**Keywords:** brucellosis diagnosis, *Brucella suis* S2, qPCR, digital PCR, nucleic acid secondary structure, SNP locus

## Abstract

**Background:**

Brucellosis, a zoonotic bacterial infection caused by *Brucella* species, exhibits a global distribution. The *Brucella* S2 vaccine strain is known to cause brucellosis. Current serological antibody assays cannot distinguish between infections caused by the S2 strain and those caused by wild-type *Brucella*.

**Objective:**

To develop a diagnostic method capable of specifically detecting S2 vaccine strain infections.

**Methods:**

Two probes were designed targeting single nucleotide polymorphism (SNP) loci upstream of the *sugar ABC* gene; quantitative polymerase chain reaction (qPCR) and droplet digital polymerase chain reaction (ddPCR) methods were established. The performances of these methods were evaluated. The transient stem-loop structure of the DNA template was predicted, and the impact of probe overlap with the transient stem-loop structure on detection sensitivity was analyzed. Clinical applicability was assessed using 50 blood samples from brucellosis patients.

**Results:**

Both types of methods demonstrated high specificity. However, MGB-SNPdd showed greater sensitivity than other detection methods. Reduction of overlap between the probe sequence and the transient stem-loop structure enhanced detection sensitivity. In the clinical applicability analysis, ddPCR methods exhibited higher rates of S2 vaccine strain detection compared with qPCR methods.

**Conclusion:**

SNP-based ddPCR methods demonstrate higher sensitivity than qPCR methods and enable specific detection of brucellosis caused by the S2 vaccine strain. Reduction of probe overlap with the transient stem-loop structure improves detection sensitivity, providing valuable insights for enhanced PCR amplification efficiency.

## Highlights

- An SNP-based ddPCR method was developed to detect *Brucella* S2 vaccine strain infections.
- The method enables laboratory identification of occupational brucellosis and detection of S2 vaccine strain transmission in livestock, preventing economic losses from misidentification and unnecessary culling.
- Reduction of overlap between probe sequences and transient stem-loop structures during DNA amplification enhances detection sensitivity.

## 1 Introduction

Brucellosis, a zoonotic bacterial infection caused by *Brucella* species, remains an important global public health problem (1). Each year, over 500,000 new cases are reported, with the highest incidence rates in South America, Africa, the Middle East, and Asia (2). In recent years, brucellosis has become increasingly endemic in China. According to data from the Chinese Center for Disease Control and Prevention, the incidence of brucellosis in China displayed an overall upward trend from 2006 to 2021. Although a temporary decline was evident between 2016 and 2018, incidence rates increased each year from 2019 to 2021. In 2021, brucellosis cases were reported in 31 provinces and autonomous regions, with a total of 69,767 cases and a record-high incidence rate of 4.95 cases per 100,000 population. These findings underscore the growing public health burden posed by the disease (3).

Vaccination remains a cornerstone of brucellosis prevention and control in livestock. Several vaccines are widely used, including *Brucella melitensis* M5, *B. melitensis* strain Rev1, and *Brucella abortus* strains 19 and RB51. Although these vaccines provide varying levels of protection, their overall effectiveness has been suboptimal (4). The *B. suis* strain S2 vaccine, developed in 1952, offers strong protection but requires high doses for immunization and carries considerable toxicity risks after administration (4–7). The high doses of vaccine can result in infections among farm workers or laboratory staff and may trigger innate immune responses that exacerbate inflammation (4, 6, 8). Previous studies analyzing brucellosis cases and vaccine-related infections in China from 2006 to 2019 have shown that symptoms resulting from S2 vaccine strain infections are indistinguishable from those caused by natural infections with wild-type strains. Such symptoms include fever, night sweats, general malaise, and fatigue, indicating the pathogenic potential of the S2 vaccine strain in humans (9, 10). In 2017, an outbreak of brucellosis occurred in Tianzhu County, Gansu Province, China, involving 206 animal disease control personnel who participated in S2 vaccine immunization efforts from November to December 2016. Among these personnel, 51 exhibited S2 vaccine antibody positivity, representing an infection rate of 24.8%. The primary causes of infection were improper vaccination procedures, inadequate biosafety measures, limited awareness of the pathogenicity of the S2 vaccine strain, insufficient personal protective equipment, and the absence of a robust emergency response plan (11).

In the Inner Mongolia Autonomous Region, newly reported brucellosis cases constitute approximately 40% of the national total. Tongliao City, a key pastoral area in eastern Inner Mongolia, exhibited an incidence rate of 9.22–38.56 cases per 100,000 people between 2007 and 2017 (12). In this high-incidence region, the S2 vaccine strain is the primary vaccine used to immunize domestic animals such as cattle, sheep, and pigs (13). During the development, production, and administration of the S2 vaccine, personnel—particularly those involved in animal disease control—face an inevitable risk of infection. Because of the high genetic similarity between the S2 vaccine strain and wild-type *Brucella* strains, serological tests cannot effectively differentiate between antibody responses induced by the S2 vaccine strain and those caused by natural infections with wild-type strains; thus, it is difficult to distinguish S2 vaccine-induced artificial immunity from naturally acquired immunity. Worldwide (including China), there is a lack of accurate, rapid, and easily implemented laboratory diagnostic methods for identifying S2 vaccine strain infections, particularly in primary healthcare settings. Thus, a novel diagnostic method is needed for effective detection of S2 vaccine strain infections. Such a method would address the limitations of current serological testing, enabling clear differentiation between S2 vaccine strain infections and natural wild-type *Brucella* infections. It would also provide a valuable diagnostic tool for identifying occupational brucellosis in animal disease control personnel.

This study aimed to achieve precise detection of S2 vaccine strain infections by designing TaqMan-MGB and Affinity Plus probes targeting single nucleotide polymorphism (SNP) loci upstream of the *sugar ABC* gene (NCBI Reference Sequence: NZ_CP025819.1 REGION: 246535.247854). Quantitative polymerase chain reaction (qPCR) and droplet digital polymerase chain reaction (ddPCR) methods were developed and rigorously evaluated. The transient stem-loop structure of the DNA template was predicted, and the impact of probe overlap with the transient stem-loop structure on detection sensitivity was analyzed. Clinical applicability was assessed using 50 blood samples from brucellosis patients. The study successfully established a highly sensitive ddPCR method for specific diagnosis of *Brucella* S2 vaccine strain infections, utilizing SNP loci upstream of the *sugar ABC* gene. Furthermore, the study revealed that the development of PCR-based detection methods requires consideration of the effects of probe and primer secondary structures on amplification sensitivity, as well as the influence of transient stem-loop structures formed by the DNA template. These findings provide valuable insights for enhancing PCR amplification efficiency in future diagnostic applications.

## 2 Materials and methods

### 2.1 Genome of experimental strains

**Figure 1:**
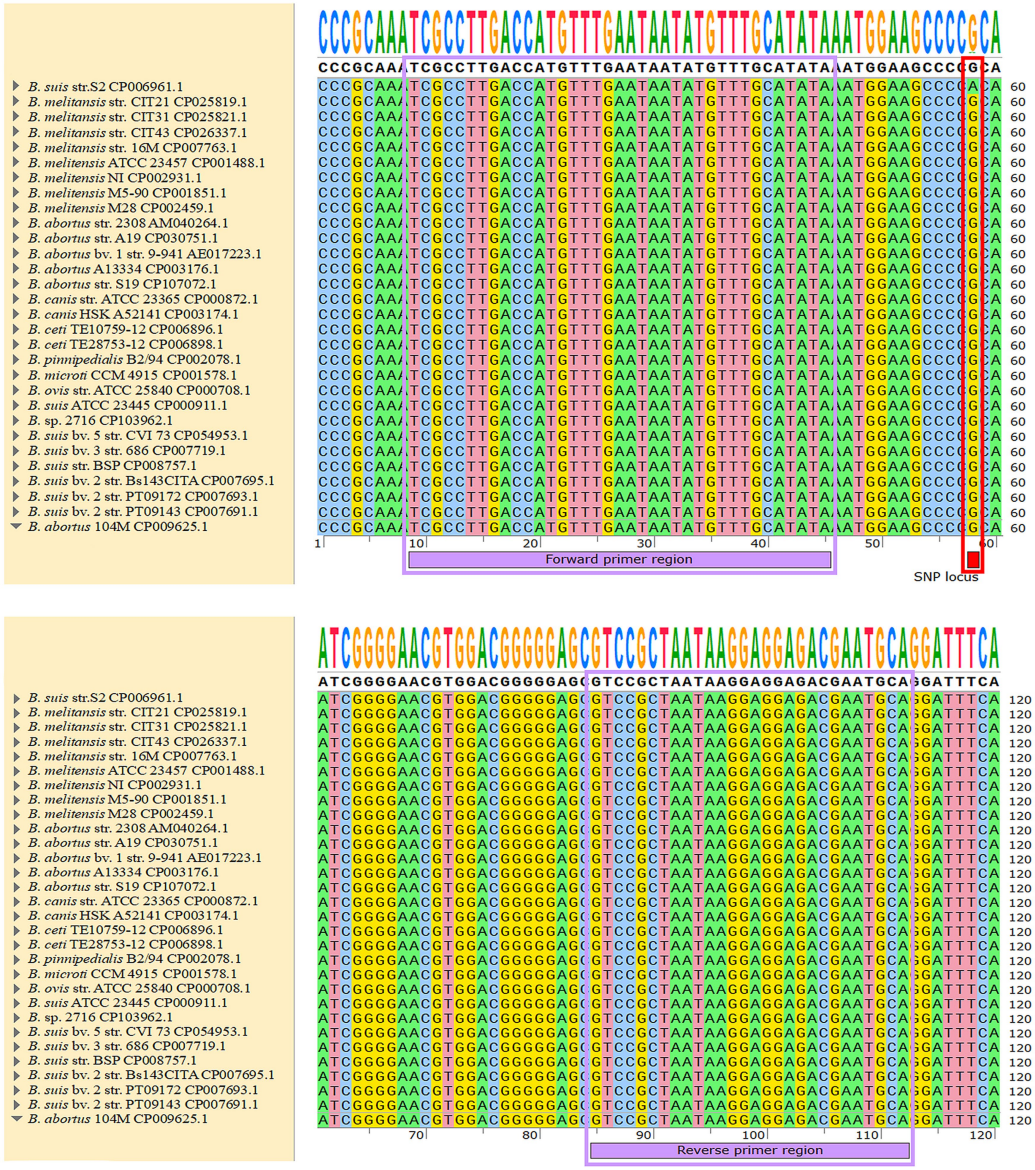
SNP loci of 30 *Brucella* strains. The *sugar ABC* gene and upstream sequences of the 30 *Brucella* strains listed in Table 1 were compared. A mutation was identified at the 50th base upstream of the *sugar ABC* gene in the S2 vaccine strain, resulting in a distinct SNP site compared with the other 29 *Brucella* strains. With the exception of the S2 vaccine strain, the *sugar ABC* gene and upstream sequences were identical among the remaining strains. Red marks indicate SNP sites, purple marks indicate primer binding regions, and strain names are listed on the left side of the figure. The analyzed strains include vaccine strains, swine *Brucella* strains, and other rare *Brucella* strains.

During the initial phase of the study, genomic sequences of 22 *Brucella* wild-type strains and vaccine strains, including the *Brucella* S2 vaccine strain (GenBank accession number CP006961.1) were analyzed using the National Center for Biotechnology Information (NCBI) database. Sequence comparison identified five SNP loci specific to the S2 vaccine strain. In this study, in addition to the original 22 strains, *Brucella* strains isolated from pigs and the 104M vaccine strain were included, resulting in a total of 30 *Brucella* strains for comparative analysis. The strain names and GenBank accession numbers are listed in Table 1; sequence comparison results are presented in Figure 1. As illustrated in Figure 1, sequences from the 29 non-S2 strains were highly conserved. *Brucella* wild-type strains prevalent in Tongliao, Inner Mongolia, were sequenced and deposited in GenBank. These strains included *B. melitensis* str. CIT21 (GenBank accession numbers CP025819.1, CP025820.1), *B. melitensis* str. CIT31 (GenBank accession numbers CP025821.1, CP025822.1), and *B. melitensis* str. CIT43 (GenBank accession number CP026337) (14–18). The *sugar ABC* gene and its upstream sequences were identical across these three wild-type strains. For subsequent experiments, the wild-type strain CIT21 (GenBank accession number CP025819.1) was selected as the positive control. The *sugar ABC* gene sequences of the S2 vaccine strain and CIT21 wild-type strain, along with 200 upstream bases, were cloned into the pUC57 vector to serve as plasmid standards for subsequent sensitivity and specificity analyses. Genomic DNA samples from the *Brucella* S2 vaccine strain, CIT21 wild-type strain, *Salmonella* Typhimurium, *Escherichia coli*, and *Staphylococcus aureus* were obtained from the Key Laboratory of Zoonotic Disease Control and Prevention at the Autonomous Region University; all samples were stored at −20°C. Genomic DNA from *Salmonella* Typhimurium, *E. coli*, and *S. aureus* was used to verify the specificity of the detection method. Prior to these experiments, the *sugar ABC* gene and the 200 bp upstream regions of both the *Brucella* S2 vaccine and CIT21 wild-type strains were compared using the NCBI BLAST tool. No sequence homology was observed with the genomes of *Salmonella* Typhimurium, *E. coli*, or *S. aureus*.

**Table 1:** Names, GenBank accession numbers, and SNP loci information of 30 *Brucella* strains.

Bacteria species	Accession number	*Sugar ABC* SNP locus	Source
*B. suis* str. S2	CP006961.1	A	Qingdao, Shandong, 266032, China
*B. melitansis* str. CIT21	CP025819.1	G	Tongliao City, 028000, China
*B. melitansis* str. CIT31	CP025821.1	G	Tongliao City, 028000, China
*B. melitansis* str. CIT43	CP026337.1	G	Tongliao City, 028000, China
*B. melitansis* str. 16M	CP007763.1	G	Los Alamos, NM 87545, United States
*B. melitensis* ATCC 23457	CP001488.1	G	Blacksburg, VA 24061, United States
*B. melitensis* NI	CP002931.1	G	Beijing, 100094, China
*B. melitensis* M5-90	CP001851.1	G	Harbin, 150001, China
*B. melitensis* M28	CP002459.1	G	Harbin, 150001, China
*B. abortus* str. 2308	AM040264.1	G	Oak Ridge, TN 37831, United States
*B. abortus* str. A19	CP030751.1	G	Hohhot, 010018, China
*B. abortus* bv. 1 str. 9-941	AE017223.1	G	Ames, IA 50010, United States
*B. abortus* A13334	CP003176.1	G	Anyang, Kyunggi-do, Republic of Korea
*B. abortus* str. S19	CP107072.1	G	Hohhot, 010018, China
*B. canis* str. ATCC 23365	CP000872.1	G	Blacksburg, VA 24061, United States
*B. canis* HSK A52141	CP003174.1	G	Anyang, Kyunggi-do, Republic of Korea
*B. ceti* TE10759-12	CP006896.1	G	Pula, Cagliari 09010, Italy
*B. ceti* TE28753-12	CP006898.1	G	Pula, Cagliari 09010, Italy
*B. pinnipedialis* B2/94	CP002078.1	G	Marseille cedex 09 FR-13288, France
*B. microti* CCM 4915	CP001578.1	G	Marseille cedex 09, France
*B. ovis* str. ATCC 25840	CP000708.1	G	Rockville, MD 20850, United States
*B. suis* ATCC 23445	CP000911.1	G	Blacksburg, VA 24061, United States
*B.* sp. 2716	CP103962.1	G	Vienna 1210, Austria
*B. suis* bv. 5 str. CVI 73	CP054953.1	G	Zagreb 10000, Croatia
*B. suis* bv. 3 str. 686	CP007719.1	G	Los Alamos, NM 87545, United States
*B. suis* str. BSP	CP008757.1	G	Los Alamos, NM 87545, United States
*B. suis* bv. 2 str. Bs143CITA	CP007695.1	G	Campo Grande, Lisboa 1749-016, Portugal
*B. suis* bv. 2 str. PT09172	CP007693.1	G	Campo Grande, Lisboa 1749-016, Portugal
*B. suis* bv. 2 str. PT09143	CP007691.1	G	Campo Grande, Lisboa 1749-016, Portugal
*B. abortus* 104M	CP009625.1	G	Beijing, 100071, China

### 2.2 Extraction of DNA from clinical samples

The diagnostic criteria established by the Medical Emergency Department of the National Health Commission of the People’s Republic of China, titled “Diagnosis and Treatment Plan for Brucellosis (2023 Edition),” were adopted as inclusion criteria for patients with brucellosis in this study. The specific criteria were as follows: patients exhibited clinical manifestations of brucellosis or a positive result in the Rose Bengal test (RBT), along with a serum tube agglutination test (SAT) titer of 1:100++ or higher; for those with clinical symptoms persisting for over 1 year, the SAT titer was 1:50++ or higher.

Blood samples from 50 brucellosis patients diagnosed at a hospital in Tongliao City were collected for *Brucella* DNA extraction using the serum blood cell synchronous detection method (Patent No. US-11505834-B2) (19). The protocol was performed in accordance with instructions outlined in the patent (20). This study was reviewed and approved by the ethics committee (approval number: NM-LL-2024-09-25-01).

### 2.3 Primer and probe design

The *sugar ABC* gene sequences of the *Brucella* S2 vaccine strain and 22 other *Brucella* strains were utilized for sequence alignment of 120 upstream bases using SnapGene software (version 7.0.2, United States). As shown in Figure 1, the *sugar ABC* gene sequences of the 22 strains, excluding the S2 vaccine strain, were identical throughout the upstream 120 bases. An SNP at the 50th base of the *sugar ABC* gene sequence clearly distinguished the *Brucella* S2 vaccine strain from the other strains (see Figure 1). Primers targeting this region were predicted to ensure specific detection of the S2 vaccine strain. Primers and probes for TaqMan-MGB and Affinity Plus assays were designed using Primer Premier 6.0 (Canada) and Primer Express 3.0.1 (United States); their sequences are listed in Table 2. The TaqMan-MGB probe and primer sequences were derived from our previous work (5) and synthesized by Sangon Biotech (Shanghai, China). The Affinity Plus probe and primers were synthesized by Integrated DNA Technologies (United States). qPCR and ddPCR methods using the TaqMan-MGB probe were named MGB-SNPq and MGB-SNPdd, respectively. Corresponding methods using the Affinity Plus probe were named LNA-SNPq and LNA-SNPdd, respectively.

**Table 2:** Gene targets, primers, and allele-specific probes used in this study.

Probe type	Primer sequence (5′ → 3′)	Length (bp)	Probe sequence	Label
MGB probe	F-TCGCCTTGACCATGTTTGAA	104	FAM-ATATAAATGGAAGCCCC(A)CA-MGB	S2 vaccine strain
	R-TGCATTCGTCTCCTCCTTATTAGC		VIC-CATATAAATGGAAGCCCC(G)CA-MGB	Other 29 strains
Affinity Plus probe	F-ACCATGTTTGAATAATATGTTTGCATATA	88	FAM-AAGCCCC(A)CAATC-IABkFQ	S2 vaccine strain
	R-TCTCCTCCTTATTAGCGGAC		VIC-CCCC(G)CAATCG-IABkFQ	Other 29 strains

### 2.4 Prediction of DNA template secondary structure

To examine the effect of DNA template secondary structures on amplification efficiency during PCR, secondary structures were predicted under identical concentrations of sodium and magnesium ions with a consistent temperature. These predictions were performed using the “mfold Web” server (Rensselaer Polytechnic Institute, United States) (21).

### 2.5 qPCR amplification conditions

For MGB-SNPq and LNA-SNPq detection, a 20-μL reaction mixture was prepared containing the following components: 10 μL Premix Ex Taq (TaKaRa, Code No. RR390A), 0.4 μL primers (final concentration 200 nM), 0.4 μL probes (final concentration 200 nM), 2 μL ROX reference dye, 2 μL DNA template, and double-distilled water (ddH_2_O) to a final volume of 20 μL. Amplification experiments were conducted on a StepOnePlus Real-Time PCR System (Thermo Fisher Scientific, United States) with the following cycling conditions: initial denaturation at 95°C for 20 s, followed by 40 cycles of denaturation at 95°C for 1 s and annealing/extension at 60°C for 20 s.

### 2.6 ddPCR amplification conditions

For MGB-SNPdd and LNA-SNPdd detection, a 30-μL reaction mixture was prepared containing the following components: 15 μL Probe dPCR SuperMix (TARGETING ONE Biotech Co., Ltd., China), 1 μL DNA template, 1.2 μL primers (final concentration 400 nM), 0.6 μL probe (final concentration 200 nM), and ddH_2_O to a final volume of 30 μL. The mixture was dispensed into the wells of a droplet generation chip, and 180 μL of droplet generation oil were added to the oil wells. The chip was loaded into the Drop Marker M1 sample preparation instrument (TARGETING ONE) for droplet generation. Amplification experiments were performed using an A300 PCR Amplifier (Long Gene, Hangzhou, China) with the following cycling conditions: pre-denaturation at 95°C for 10 min, followed by 40 cycles of denaturation at 95°C for 30 s and annealing/extension at 60°C for 1 min. Fluorescence signals were detected via the Chip Reader R1 Biochip Analyzer.

### 2.7 Analyses of ddPCR and qPCR sensitivity and specificity

The sensitivities (detection limits) of qPCR and ddPCR methods were evaluated using pUC57 vector plasmid standards containing the *sugar ABC* gene sequence and 200 upstream bases from the *Brucella* S2 vaccine strain and CIT21 wild-type strain. For qPCR sensitivity testing, plasmid dilutions of the S2 vaccine strain and CIT21 wild-type strain ranging from 10^8^ to 10^0^ copies/μL were used as templates; for ddPCR sensitivity testing, plasmid dilutions ranging from 10^5^ to 10^0^ copies/μL were used as templates. Each concentration was tested in triplicate.

The copy numbers of the plasmid standards were calculated as follows:


$$\begin{array}{l} \text{Copy number}\;(\text{copies}/\mathrm{\mu L})=(\begin{array}{l} \text{Concentration}\;[\text{ng}/\mathrm{\mu L}]\times \\ 6.022\times 10^{23} \end{array})/\\ \;(\text{Bases}\times 660\times 1\times 10^{9}). \end{array}$$

The specificities of qPCR and ddPCR detection methods were assessed using DNA from *Salmonella* Typhimurium, *E. coli*, and *S. aureus* as templates for bacterial detection. The S2 vaccine strain and CIT21 wild-type strain served as positive controls (PCs), whereas ddH_2_O comprised the negative control (NC). Specificity experiments were performed using both the TaqMan-MGB and Affinity Plus probes in the qPCR and ddPCR methods.

### 2.8 Detection of *Brucella* DNA in brucellosis patients

To compare the detection capabilities of qPCR and ddPCR and assess the clinical applicability of the developed methods, MGB-SNPq, MGB-SNPdd, LNA-SNPq, and LNA-SNPdd were utilized to detect *Brucella* DNA in blood samples from 50 patients diagnosed with brucellosis. Concurrently, our existing recombinase polymerase amplification (RPA)—clustered regularly interspaced short palindromic repeats (CRISPR)/CRISPR-associated protein (Cas)12a real-time fluorescence quantitative method (22) was used as a reference for parallel detection in the same samples. Receiver operating characteristic (ROC) curve analysis was conducted for the established MGB-SNPq, MGB-SNPdd, LNA-SNPq, and LNA-SNPdd methods to evaluate their diagnostic performance.

## 3 Results

### 3.1 Predicted template secondary structures

**Figure 2:**
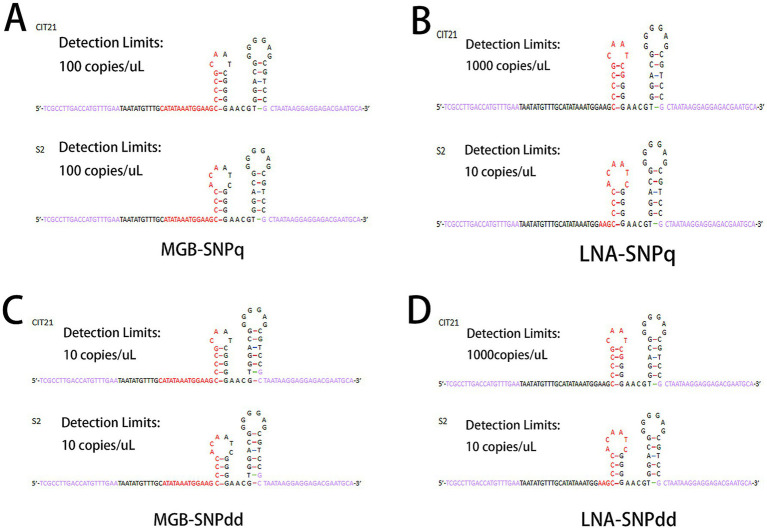
Predicted transient stem-loop structures. Under identical reaction conditions and within the same reaction system, the transient stem-loop structures of the template DNA were predicted using the “mfold Web” server. In the predicted structures, primer sequences are shown in purple and probe sequences are shown in red. The probe overlap rates with the predicted stem-loop structure were 33.3% for the CIT21 wild-type strain and 35% for the S2 vaccine strain in the MGB-SNPq and MGB-SNPdd assays **(A,C)**. For the LNA-SNPq and LNA-SNPdd assays, the overlap rates were 100% for the CIT21 wild-type strain and 76.9% for the S2 vaccine strain **(B,D)**. The detection limits of the MGB-SNPq method were 100 copies/μL for both the CIT21 wild-type and S2 vaccine strains. For the MGB-SNPdd method, these detection limits were both 10 copies/μL. The detection limits of the LNA-SNPq method were 1,000 copies/μL for the CIT21 wild-type strain and 10 copies/μL for the S2 vaccine strain. For the LNA-SNPdd method, these detection limits were 1,000 copies/μL and 10 copies/μL, respectively.

The transient stem-loop structure of each DNA template was predicted using the “mfold Web” server, with primer and probe positions annotated as shown in Figure 2. The predicted stem-loop structures for the S2 vaccine strain and CIT21 wild-type strain included annotations for detection limits, with primer and probe regions highlighted in purple and red, respectively. The number of base overlaps between each probe sequence and the transient stem-loop structure of the corresponding template was calculated. Regarding the MGB-SNPq and MGB-SNPdd probes, rates of overlap with the transient stem-loop structure were 33.3% for the CIT21 wild-type strain and 35% for the S2 vaccine strain (Figures 2A,C). Concerning the LNA-SNPq and LNA-SNPdd probes, rates of overlap with the transient stem-loop structure were 100% for the CIT21 wild-type strain and 76.9% for S2 vaccine strain (Figures 2B,D).

### 3.2 Validation of ddPCR and qPCR primer and probe specificity

**Figure 3:**
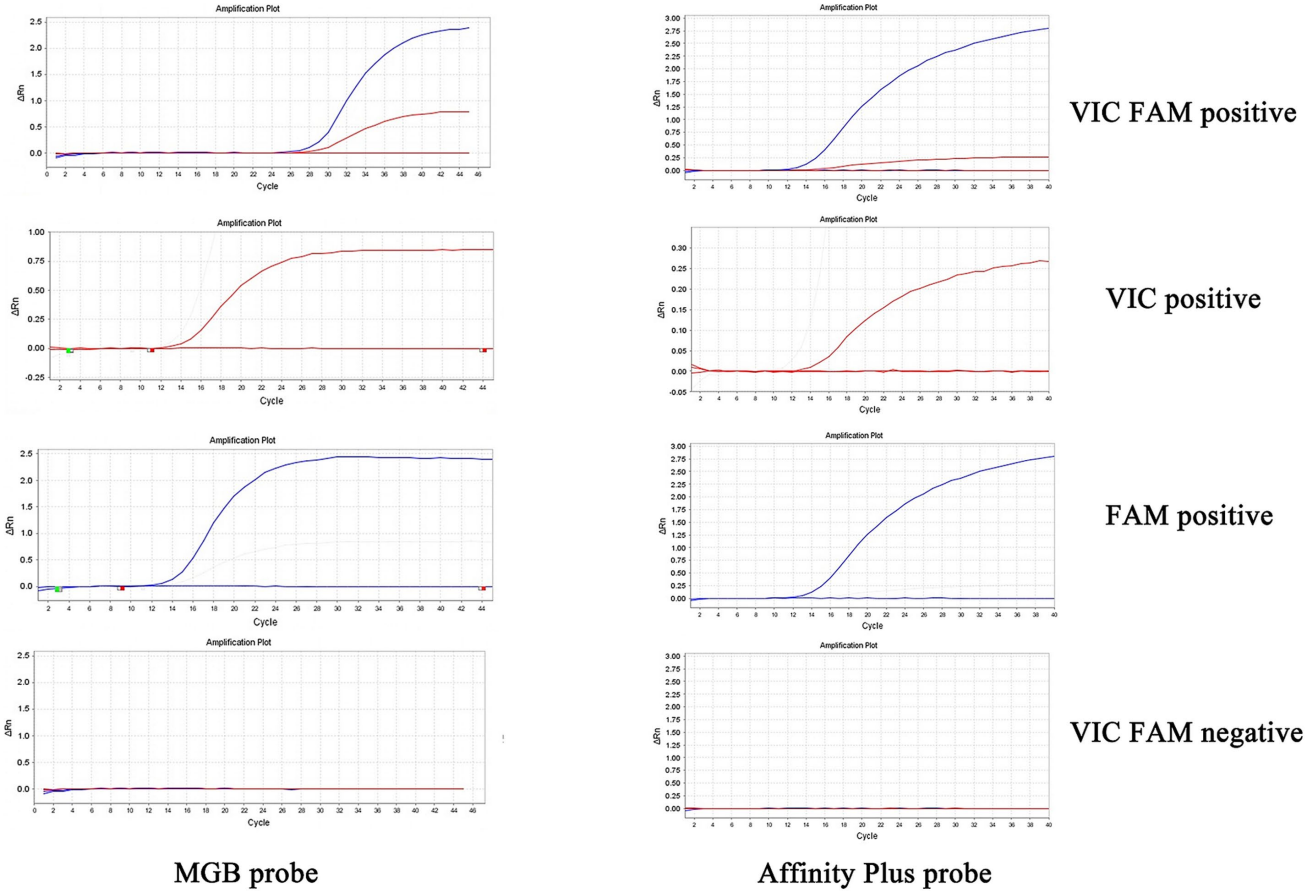
qPCR detection of strains. In this figure, the FAM channel is represented by the blue curve, indicating detection of the S2 vaccine strain, whereas the VIC channel is denoted by the red curve, indicating detection of the wild-type CIT21 strain. VIC/FAM dual-positive detection targets both the S2 vaccine and wild-type CIT21 strains. VIC-positive detection specifically targets the wild-type CIT21 strain; FAM-positive detection targets the S2 vaccine strain. VIC/FAM-negative detection targets ddH_2_O as a negative control to confirm primer and probe specificity. The results demonstrated that the MGB-SNPq and LNA-SNPq methods both accurately detect the S2 vaccine strain and clearly distinguish it from the wild-type CIT21 strain, thus verifying primer and probe specificity. No non-specific amplification was observed with any primer-probe combinations.

**Figure 4:**
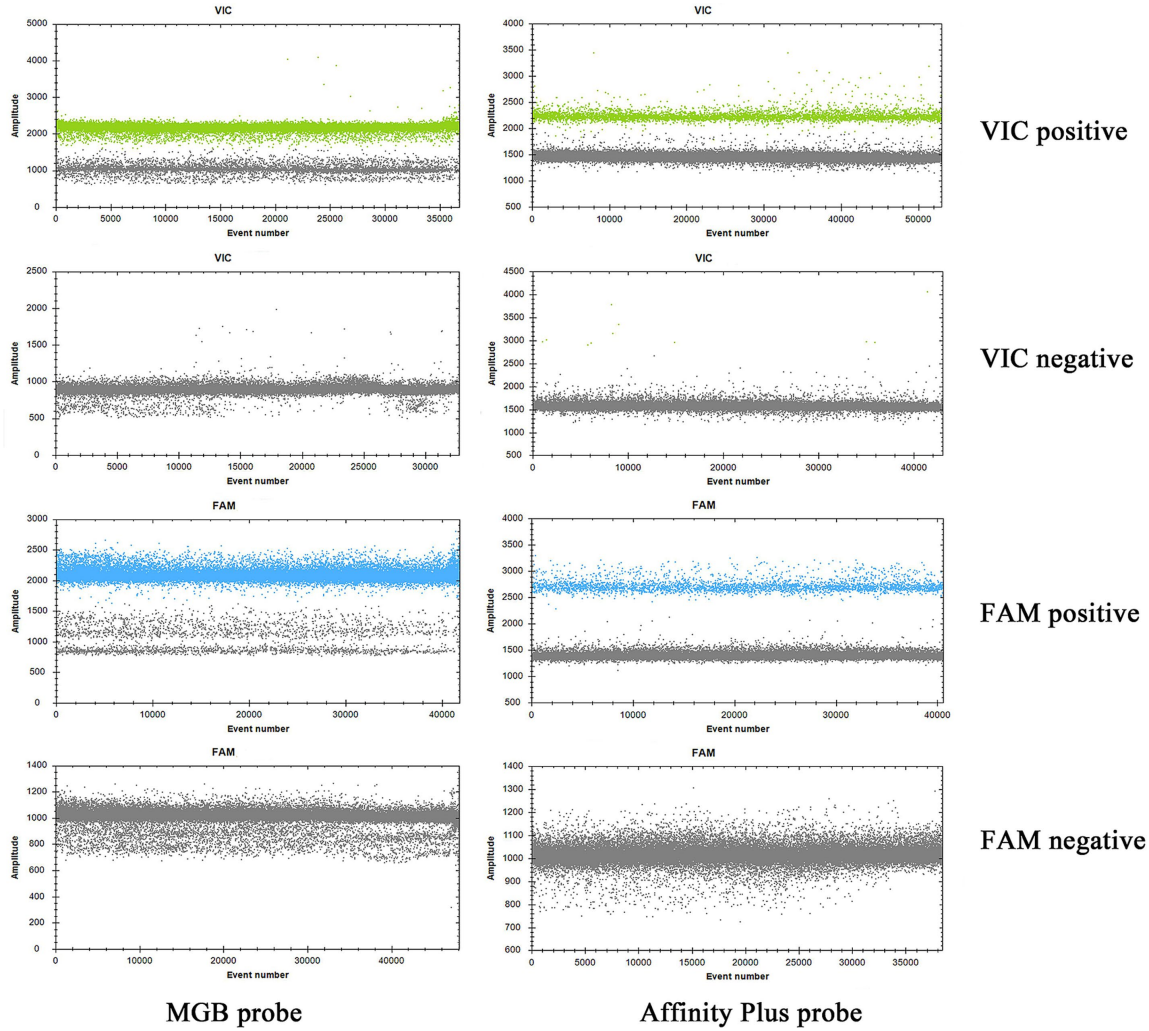
ddPCR detection of strains. In this figure, the FAM channel is shown in blue for detection of the S2 vaccine strain, and the VIC channel is shown in green for detection of the CIT21 wild-type strain. VIC-positive detection targets the CIT21 wild-type strain, whereas FAM-positive detection targets the S2 vaccine strain. VIC/FAM-negative detection targets ddH_2_O as a negative control to confirm primer and probe specificity. The results demonstrated that the MGB-SNPdd and LNA-SNPdd methods accurately detect both the S2 vaccine and CIT21 wild-type strains, thus verifying primer and probe specificity. No non-specific amplification was observed with any primer-probe combinations.

The specificities of primers and probes were validated for the S2 vaccine strain and CIT21 wild-type strain using both MGB and Affinity Plus probes in ddPCR and qPCR assays. In the qPCR assay, the primers and probes demonstrated high specificity—no non-specific amplification was observed for any primer-probe pair. Each FAM-labeled probe appeared blue, and each VIC-labeled probe appeared red. The assay accurately detected the S2 vaccine strain and effectively differentiated it from the CIT21 wild-type strain (Figure 3). In the ddPCR assay, the primers and probes also showed high specificity—no non-specific amplification was detected for any primer-probe pair. Each FAM-labeled probe appeared blue, and each VIC-labeled probe appeared green. Positive and negative droplet clusters were clearly distinguishable, enabling precise detection of both the S2 vaccine strain and the CIT21 wild-type strain (Figure 4).

### 3.3 Evaluation of qPCR and ddPCR sensitivity

**Figure 5:**
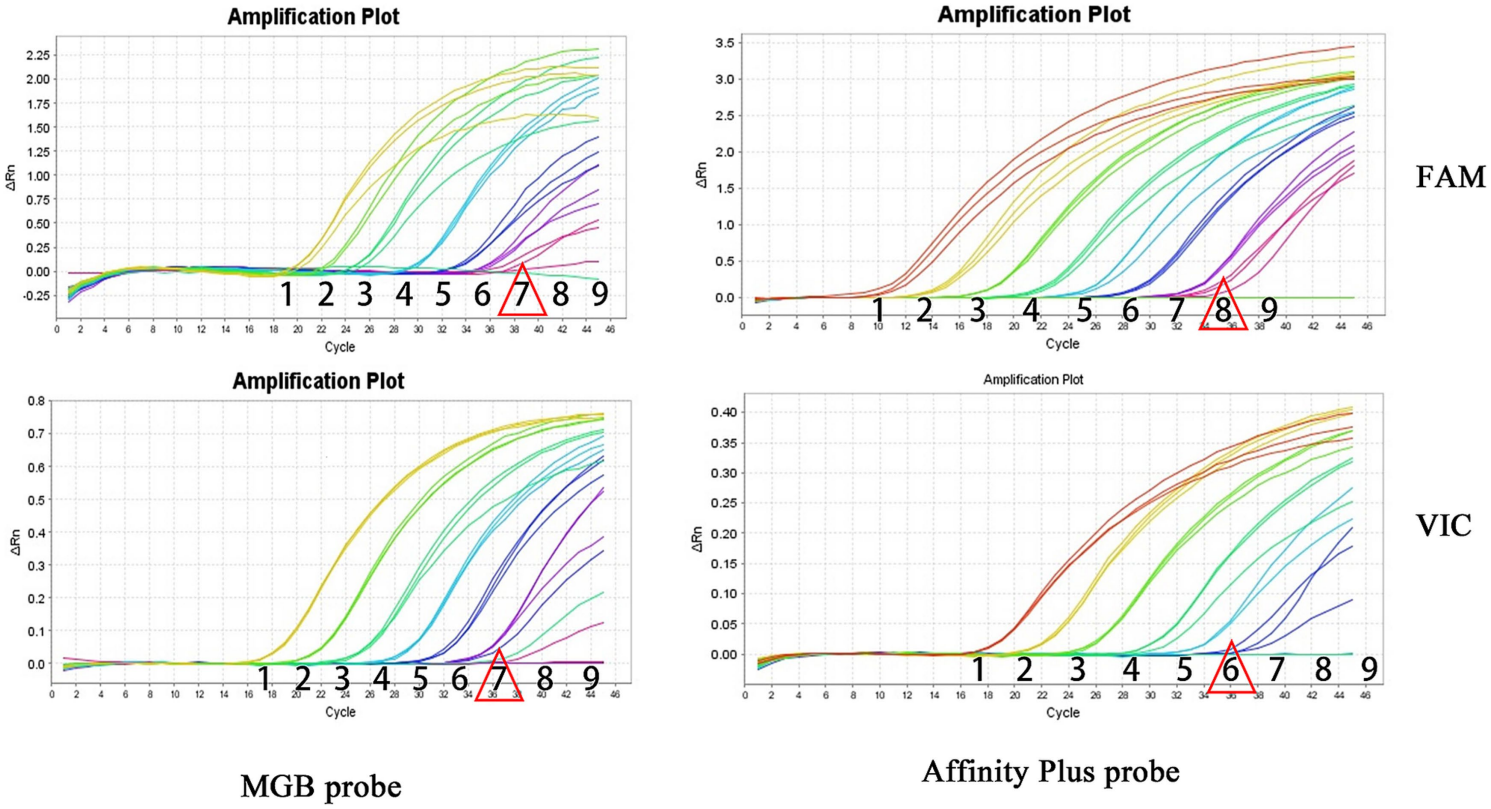
qPCR detection sensitivity. In this figure, numbers 1–9 correspond to a gradient of template concentrations ranging from 10^8^ to 10^0^ copies/μL. The FAM channel was used to detect the S2 vaccine strain, whereas the VIC channel was used to detect the CIT21 wild-type strain. Each concentration was tested in triplicate. Red triangles indicate the lowest concentration at which amplification was observed. Using the MGB-SNPq detection method, the S2 vaccine and CIT21 wild-type strains both were detected in sample 7, corresponding to a concentration of 10^2^ copies/μL. Therefore, the detection sensitivity for both strains was 100 copies/μL. Using the LNA-SNPq detection method, the S2 vaccine strain was detected in sample 8, corresponding to a concentration of 10^1^ copies/μL; the CIT21 wild-type strain was detected in sample 6, corresponding to a concentration of 10^3^ copies/μL. Therefore, the detection sensitivity for the S2 vaccine strain was 10 copies/μL; for the CIT21 wild-type strain, it was 1,000 copies/μL.

**Figure 6:**
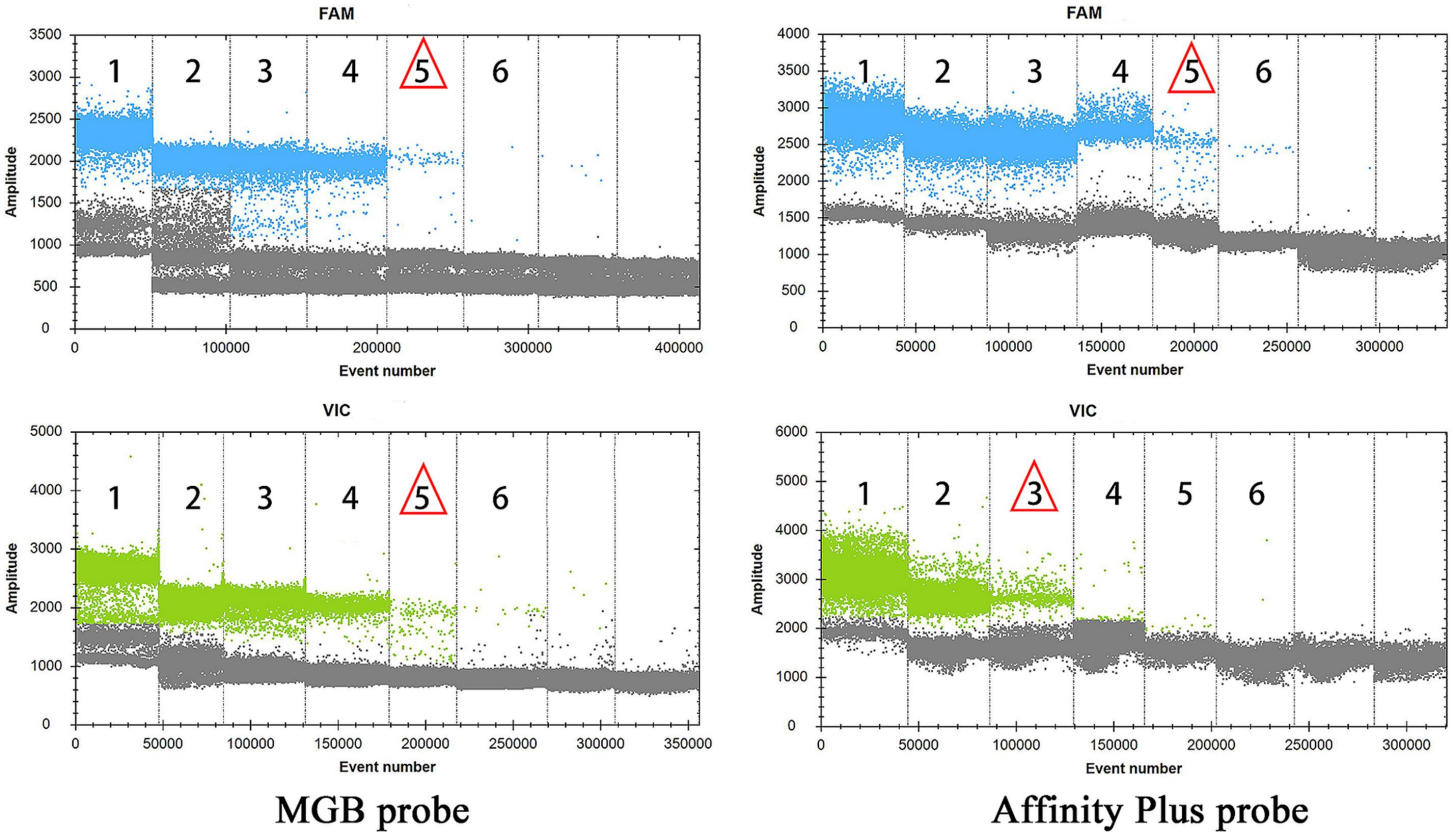
ddPCR detection sensitivity. In this figure, numbers 1–6 correspond to a gradient of template concentrations ranging from 10^5^ to 10^0^ copies/μL. The FAM channel (blue) was used to detect the S2 vaccine strain, whereas the VIC channel (green) was used to detect the CIT21 wild-type strain. Red triangles indicate the lowest concentration at which amplification was observed. Using the MGB-SNPdd detection method, the lowest detectable concentrations for the S2 vaccine and CIT21 wild-type strains were 10^1^ copies/μL, corresponding to a detection sensitivity of 10 copies/μL. Using the LNA-SNPdd detection method, the lowest detectable concentrations were 10^1^ copies/μL for the S2 vaccine strain and 10^3^ copies/μL for the CIT21 wild-type strain, corresponding to respective detection sensitivities of 10 copies/μL and 1,000 copies/μL.

Sensitivity experiments for qPCR and ddPCR generated amplification curves (Figure 5) and droplet titration plots (Figure 6). MGB-SNPq exhibited a sensitivity of 100 copies/μL for both the S2 vaccine strain and the CIT21 wild-type strain. MGB-SNPdd showed a sensitivity of 10 copies/μL for both strains. LNA-SNPq exhibited sensitivities of 10 copies/μL for the S2 vaccine strain and 1,000 copies/μL for the CIT21 wild-type strain. LNA-SNPdd showed sensitivities of 10 copies/μL for the S2 vaccine strain and 1,000 copies/μL for the CIT21 wild-type strain.

Among the detection methods, MGB-SNPdd demonstrated the highest sensitivity (detection limit of 10 copies/μL for both the S2 vaccine strain and CIT21 wild-type strain), as summarized in Table 3.

**Table 3:** qPCR and ddPCR detection limits.

Gene name	Probe type	qPCR detection limit copies/μL		ddPCR detection limit copies/μL	
		CIT21	S2	CIT21	S2
*SugarABC*	MGB	100	100	10	10
	Affinity	1,000	10	1,000	10

### 3.4 Assessment of qPCR and ddPCR specificity

**Figure 7:**
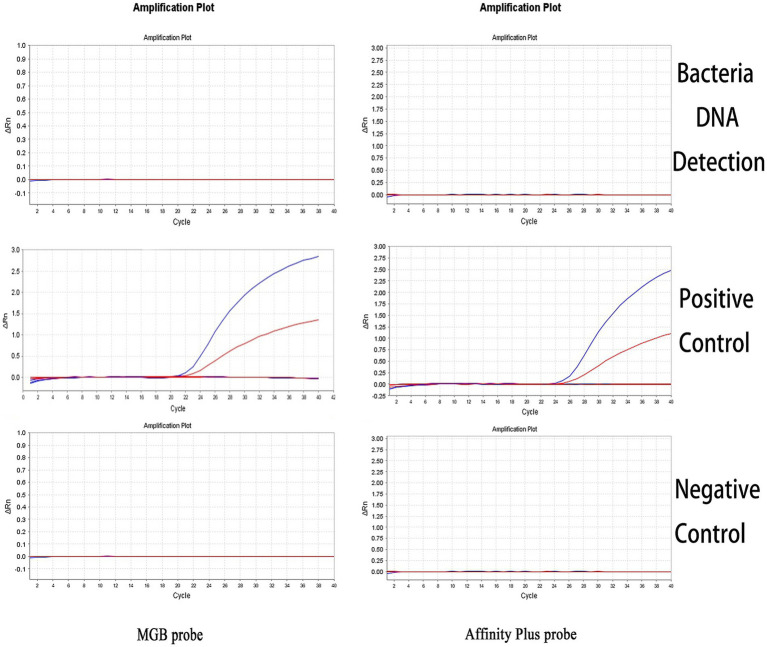
qPCR detection specificity. In the specificity analysis, the bacteria DNA detection group used DNA from *Salmonella* Typhimurium, *E. coli*, and *S. aureus* as templates. The positive control (PC) group included the S2 vaccine and CIT21 wild-type strains, whereas the negative control (NC) group used ddH_2_O. The results showed that only the PC group exhibited amplification curves in both the FAM (blue) and VIC (red) channels; no amplification was detected in the other groups. The FAM channel specifically detected the S2 vaccine strain, and the VIC channel specifically detected the CIT21 wild-type strain.

**Figure 8:**
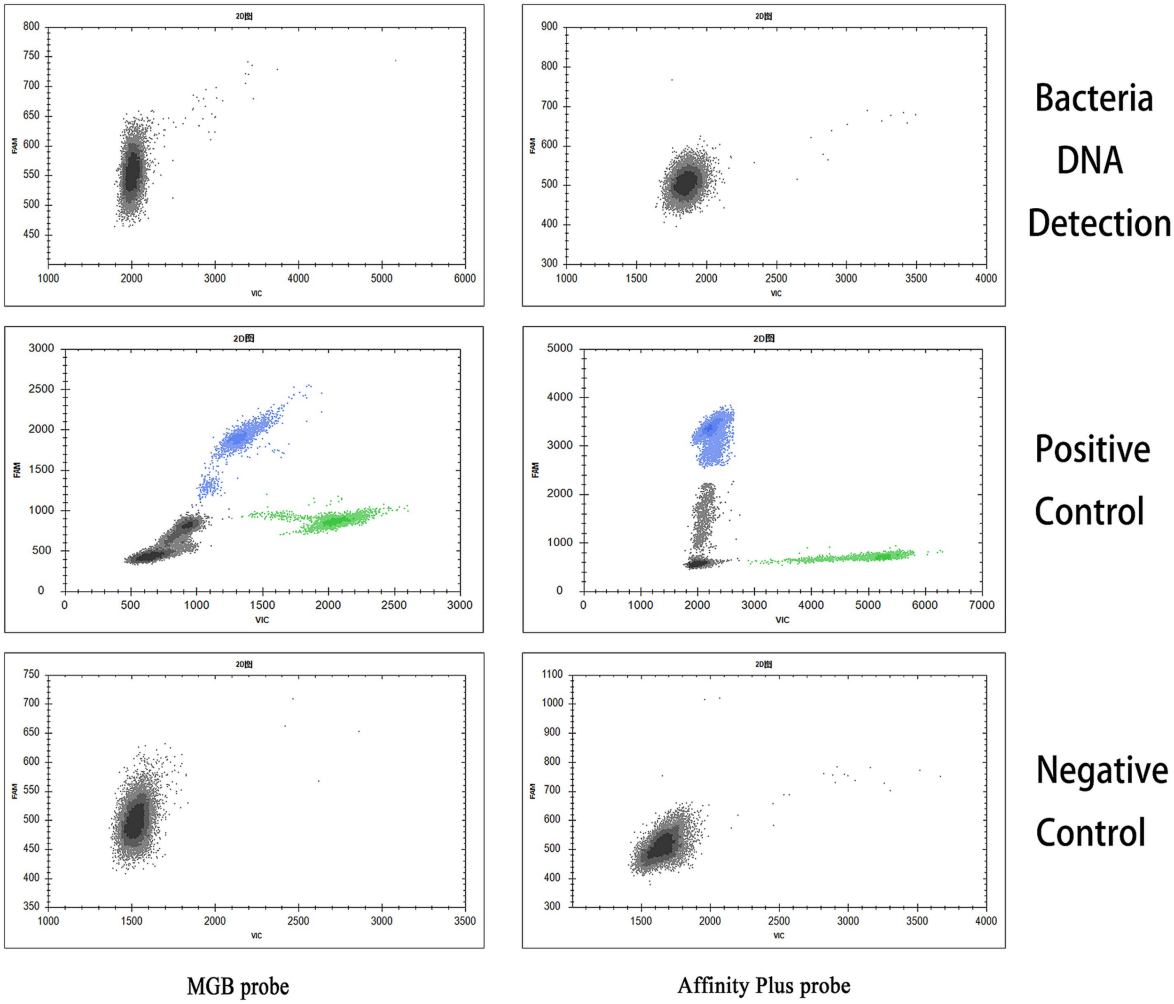
ddPCR detection specificity. In the specificity analysis, the bacteria DNA detection group used DNA from *Salmonella* Typhimurium, *E. coli*, and *S. aureus* as templates. The positive control (PC) group included the S2 vaccine and CIT21 wild-type strains, whereas the negative control (NC) group used ddH_2_O. The results showed that only the PC group exhibited amplification curves in both the FAM (blue) and VIC (green) channels; no amplification was detected in the other groups. The FAM channel specifically detected the S2 vaccine strain, and the VIC channel specifically detected the CIT21 wild-type strain.

The specificities of the qPCR and ddPCR detection methods were assessed using DNA samples from *Salmonella* Typhimurium, *E. coli*, and *S. aureus* as templates for bacterial detection. The S2 vaccine strain and CIT21 wild-type strain served as PCs, whereas ddH_2_O comprised the NC. qPCR and ddPCR methods showed no cross-reactivity (Figures 7, 8), confirming their high specificity.

### 3.5 Clinical applicability of qPCR and ddPCR methods

**Figure 9:**
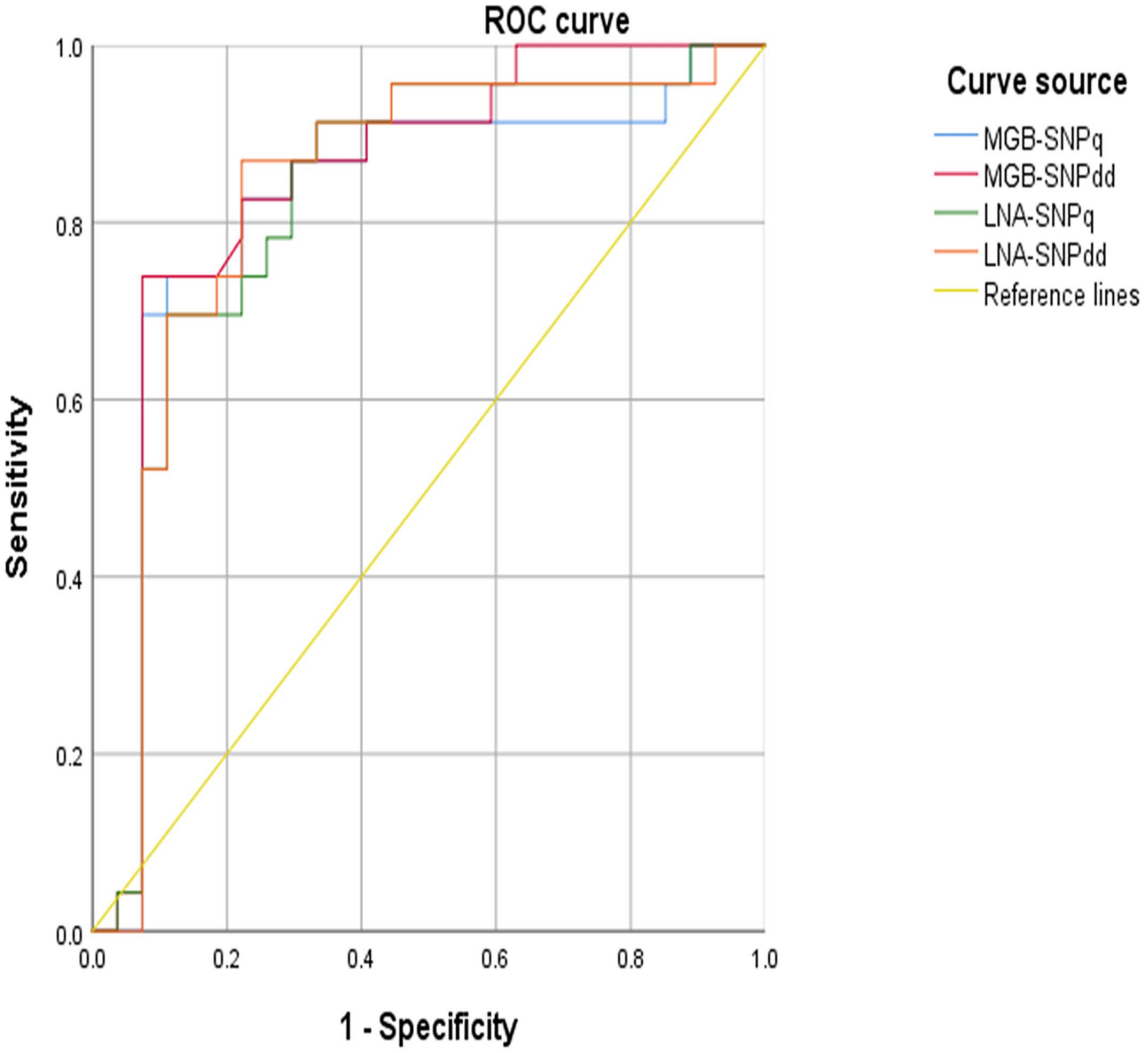
Results of ROC curve analysis. The area under the ROC curve for each detection method is as follows: MGB-SNPq, 0.818; MGB-SNPdd, 0.845; LNA-SNPq, 0.824; and LNA-SNPdd, 0.831. Among the four methods, MGB-SNPdd demonstrated the highest area under the ROC curve (0.845), followed by LNA-SNPdd (0.831), LNA-SNPq (0.824), and MGB-SNPq (0.818). Therefore, MGB-SNPdd exhibited superior diagnostic performance compared with the other three methods.

The RPA-CRISPR/Cas12a real-time fluorescence quantitative method developed by our group (22) was used in combination with the detection methods established in this study to test 50 blood samples, as well as S2-positive samples, CIT21 wild-type–positive samples, and distilled water negative controls. The results are presented in Table 4. In the *sugar ABC* MGB-SNPdd assay, VIC signals were detected in all 50 samples, whereas FAM signals were detected in two samples. The MGB-SNPq assay detected VIC fluorescence curves in 48 samples and a FAM fluorescence curve in one sample. In the *sugar ABC* LNA-SNPdd assay, VIC signals were detected in 49 samples, whereas FAM signals were detected in two samples. The LNA-SNPq assay showed VIC fluorescence curves in 48 samples and no FAM fluorescence curves. The MGB-SNPdd method yielded the highest positive detection rate based on SNPs in the upstream region of the *sugar ABC* gene (VIC: 100%, FAM: 4%), consistent with results from the RPA-CRISPR/Cas12a reference method. ROC curve analysis was conducted for the MGB-SNPq, MGB-SNPdd, LNA-SNPq, and LNA-SNPdd methods; the results are shown in Figure 9. The respective areas under the ROC curves were 0.818, 0.845, 0.824, and 0.831. The corresponding detection sensitivities—calculated using the Youden index—were 73.9, 73.9, 69.6, and 87%; the specificities were 88.9, 92.6, 88.9, and 77.8%, respectively. Among the four detection methods, MGB-SNPdd demonstrated the highest diagnostic performance.

**Table 4:** Detection of clinical samples.

Method	qPCR (*n* = 50)			ddPCR (*n* = 50)		
	VIC+ (%)	FAM+ (%)	Co-VIC FAM (%)	VIC+ (%)	FAM+ (%)	Co-VIC FAM (%)
A	48 (96%)	1 (2%)	1 (2%)	50 (100%)	2 (4%)	2 (4%)
B	48 (96%)	0	0	49 (98%)	2 (4%)	1 (2%)
C	50 (100%)	2 (4%)	2 (4%)	—	—	—

(A) MGB-SNPq and MGB-SNPdd detection based on the *sugar ABC* gene. (B) LNA-SNPq and LNA-SNPdd detection based on the *sugar ABC* gene. (C) Real-time fluorescence quantitative detection using the RPA-CRISPR/Cas12a reference method. Co-VIC FAM (%) indicates dual positivity in both the FAM and VIC channels.

## 4 Discussion

Brucellosis represents a major threat to both human and animal health, with substantial economic and social impacts in endemic regions (23, 24). In China, the disease constitutes a serious challenge; the Inner Mongolia Autonomous Region is one of the most severely impacted areas (25). Vaccination is one of the most effective strategies for the prevention and management of brucellosis. Worldwide, including in China, commonly used attenuated live vaccines include the porcine-origin *Brucella* S2 vaccine strain, ovine-origin *Brucella* M5 vaccine strain, *B. melitensis* Rev1 strain, as well as *B. abortus* strains 19 and RB51 (26, 27). In Inner Mongolia, the S2 vaccine strain is the primary vaccine used for animal immunization (13). Although the immune response induced by this vaccine is generally effective, the S2 vaccine strain shares antigens with wild-type strains. This antigenic similarity complicates the distinction between natural infections and vaccine-induced immunity when using conventional serological antibody detection methods, thereby impeding the accurate identification and management of infected animals (7). Moreover, the S2 vaccine strain poses a risk of occupational infections among animal health personnel during vaccination activities. Such risk emphasizes the need to reliably differentiate between infections caused by the vaccine strain and those caused by wild-type strains for occupational disease diagnostics. To address these issues, it is necessary to employ reliable, highly sensitive and highly specific diagnostic methods to detect the infection of the *Brucella* S2 vaccine strain.

The establishment of such methods will enable the identification of *Brucella* infections caused by the S2 vaccine strain and provide a valuable tool for the early diagnosis of brucellosis. In clinical settings, manifestations of brucellosis closely resemble those of other diseases, making accurate diagnosis challenging. Commonly used serological methods, such as the Rose Bengal test (RBT) and the tube agglutination test (SAT), often cross-react with other gram-negative bacteria. This cross-reactivity reduces sensitivity and produces inaccurate results, leading to misdiagnosis, underdiagnosis, and delayed treatment (28). Delayed diagnosis of brucellosis raises the risk of disease progression from an acute to a chronic stage, heightens the likelihood of complications, prolongs treatment durations, worsens patient outcomes, and increases economic burdens (29). Conventional bacterial culture methods, once considered the gold standard for diagnosing brucellosis, are time-consuming and lack specificity (30). PCR offers higher sensitivity and specificity compared with microbiological culture methods. Among various nucleic acid amplification techniques, challenges persist in terms of overcoming false-negative results caused by inhibitors and the low sensitivity of conventional PCR electrophoresis. qPCR, a rapid and highly specific molecular diagnostic tool, addresses some of these limitations and has been utilized to detect various microorganisms, including *Brucella*. However, qPCR accuracy largely depends on a standard curve generated with known concentrations of DNA, restricting its broader clinical application (31). Compared with qPCR, the newer ddPCR amplification method reduces inhibitor impact on PCR efficiency, maintaining accuracy and stability even in the presence of high inhibitor concentrations (32). ddPCR is particularly effective for detecting low levels of bacteria because it does not require a standard curve for absolute quantification of clinical samples (33). Although ddPCR has not been widely utilized for *Brucella* detection, the method developed in this study exhibits robust potential for accurate identification of brucellosis caused by the *Brucella* S2 vaccine strain.

Because of the rapid growth of molecular sequencing data and advancements in technological platforms, PCR-based microbial genotyping methods using SNPs have become increasingly prevalent. SNPs are single-base mutations or small insertions/deletions in nucleotide sequences that differ between homologous sequences from different individuals or within the same individual. These variations can occur in both coding and non-coding regions of genes (34, 35). SNPs in non-coding regions can affect transcription and the function of non-coding RNAs, thereby influencing gene expression. In coding regions, SNPs can be categorized as synonymous or non-synonymous (36). Synonymous SNPs do not alter the amino acid sequence because multiple codons encode the same amino acid. Although often considered “silent,” synonymous SNPs can influence gene function. For example, a novel synonymous SNP in the *PITX3* gene may enhance Parkinson’s disease risk among individuals of Chinese ethnicity (37). Non-synonymous SNPs include missense mutations, which result in an amino acid change, and nonsense mutations, which introduce a premature stop codon, leading to truncated and often non-functional protein products (38). Altered gene function can affect the infectivity and resistance profiles of pathogenic microorganisms. Notably, SNPs are superior to other markers because of their high density, biallelic nature, representativeness, and genetic stability (39). As valuable tools for genetic analysis, SNPs can be used to distinguish individuals and species, study diseases and complex traits, assess linkage disequilibrium, generate haplotype maps, and advance pharmacogenomics. A recent study demonstrated that SNP genotyping provides a rapid, cost-effective, and reliable method for monitoring severe acute respiratory syndrome coronavirus 2 (SARS-CoV-2) variants during outbreaks (40). Additionally, an SNP-based minor groove binder (MGB) PCR detection method has been developed to differentiate clearly between the *B. abortus* 104M vaccine strain and non-104M *Brucella* strains (41). However, there is currently limited information regarding SNP-based analysis methods for the *Brucella* S2 vaccine strain. The present study fills that gap by establishing a detection method for the S2 vaccine strain based on SNP sites, which will serve as a foundation for future research in this area.

Several types of probes are used for SNP analysis; in this study, we utilized TaqMan-MGB probes and Affinity Plus probes. TaqMan-MGB probes are dual-labeled probes designed with a fluorescent reporter group at the 5′ end and a non-fluorescent quencher (NFQ) combined with an MGB at the 3′ end. The NFQ substantially reduces background fluorescence, improving the signal-to-noise ratio and thus enhancing sensitivity and accuracy. The MGB, with its high affinity for the grooves of double-stranded DNA, increases the stability of the probe-template interaction. This feature enables the use of shorter probes (≥13 bases) while maintaining high mismatch discrimination and flexibility in targeting a wide range of sequences (5, 42). Affinity Plus probes incorporate up to six locked nucleic acid monomers, which improve structural stability and increase the melting temperature by more than 15°C. This adjustment allows greater flexibility in melting temperature optimization relative to MGB probes, considerably enhancing the accuracy of PCR-based allele detection and other methods that rely on differential hybridization to distinguish polymorphisms (43).

In this study, the *sugar ABC* gene, which encodes the sugar ABC transporter in *Brucella* spp., was selected as the target gene for analysis. The *sugar ABC* gene cluster encodes a bacterial sugar transport system consisting of an extracellular binding protein (Sugar A), a membrane permease protein (Sugar B), and an ATP-binding protein (Sugar C) (44, 45). The sugar ABC transporter is responsible for the uptake of various sugars, including glucose, fructose, and mannose, enabling *Brucella* to utilize host sugars as an energy source and replicate within host cells (46). Based on SNP sites located in the upstream region of this gene, TaqMan-MGB probes, Affinity Plus probes, and primers were designed to identify S2 vaccine strain infections with high accuracy, facilitating clinical detection.

In this study, MGB probes and Affinity Plus probes were used to label the S2 vaccine strain and CIT21 wild-type strain with FAM and VIC fluorescent groups, respectively. Analyses of detection performance for both methods revealed high specificity and low detection limits. As shown in Table 3, the detection limits for qPCR and ddPCR amplification using MGB probes were identical for the S2 vaccine strain and the CIT21 wild-type strain. However, when Affinity Plus probes were used, detection sensitivity was 100 times higher for the S2 vaccine strain than for the CIT21 wild-type strain. Previous studies have indicated that the secondary structure of the DNA template is often overlooked in PCR reaction design, despite its potential effects on amplification efficiency. During PCR amplification, the DNA template denatures into single strands. Due to reaction kinetics, transient stem-loop secondary structures may form within the single-stranded template; these transient structures can significantly inhibit amplification and reduce efficiency (47, 48). To explore the mechanisms underlying differences in detection limits when using the same detection method and probe type, we analyzed the transient stem-loop structures of the single-stranded templates predicted by the “mfold Web” server (Figure 2); we then recorded primer and probe positions on the predicted structures. We hypothesize that differences in detection limits result from overlap between the probe sequence and the transient stem-loop structure of the template. When the probe sequence fully overlaps with the transient stem-loop structure, the detection limit increases, reducing sensitivity. Reduction of such overlap may improve detection sensitivity, as illustrated in Figure 2. This hypothesis requires further experimental validation in future studies.

When designing PCR primers and probes, in addition to considering the impacts of the secondary structures of the primers and probes on amplification efficiency, it is important to address potential overlap between the probe sequence and transient stem-loop structures within the template. Avoidance of such overlaps or adoption of strategies to mitigate transient stem-loop structure formation in the template can improve PCR performance. Techniques such as adding dimethyl sulfoxide, betaine, or disruptors (i.e., designed oligonucleotides) can decrease the thermal stability of the template’s secondary structures, thereby enhancing amplification efficiency. This approach is particularly effective for GC-rich templates, where disruptors outperform dimethyl sulfoxide and betaine (49). By addressing the impact of transient stem-loop structures on PCR performance, the present study provides valuable insights into strategies for greater PCR amplification efficiency in future applications.

In this study, 50 clinical samples were analyzed using ddPCR and qPCR methods. The results showed that, regardless of probe differences, ddPCR achieved a higher positive detection rate than qPCR. The observed differences in performance metrics suggest that qPCR detection efficiency is considerably reduced in samples where nucleic acid amplification is inhibited by various factors. In contrast, ddPCR demonstrated distinct advantages in overcoming these limitations. Relative to qPCR, ddPCR offers greater accuracy and sensitivity in the clinical detection of brucellosis, making it a promising method with broader clinical applicability.

## 5 Conclusion

An SNP-based ddPCR method was developed to detect *Brucella* S2 vaccine strain infections. This method can be utilized for laboratory diagnostic identification of occupational brucellosis in veterinary personnel exposed to *Brucella*-infected animals. Additionally, it enables detection of S2 vaccine strain infections in livestock, preventing economic losses from misidentification and unnecessary culling. Finally, the reduction of overlap between probe sequences and transient stem-loop structures during DNA amplification enhances detection sensitivity.

## Data Availability

The original contributions presented in the study are included in the article/supplementary material, further inquiries can be directed to the corresponding authors.
